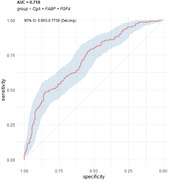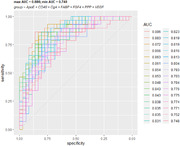# Markers of inflammation in cerebrospinal fluid can predict post‐assessment trajectory progression and classification on the ADCOMS scale

**DOI:** 10.1002/alz.088740

**Published:** 2025-01-09

**Authors:** Dave Evenden, Sofia Michopoulou, Angus Prosser, Jessica Teeling, Christopher Kipps

**Affiliations:** ^1^ University of Southampton, Southampton UK; ^2^ University Hospital Southampton NHS Foundation Trust, Southampton UK

## Abstract

**Background:**

Neuroinflammation is an integral part of Alzheimer’s Disease (AD) pathology, whereby inflammatory processes contribute to the production of amyloid‐β, the propagation of tau pathology, and neuronal loss. We recently investigated data‐driven methods for determining distinct progression trajectory groups on the ADCOMS scale. This study evaluates whether biomarkers of inflammation in cerebrospinal fluid (CSF) can predict progression rate and membership of those progression rate groups.

**Method:**

ADNI’s Biomarkers Consortium dataset provided 83 quality‐controlled CSF analyte biomarker levels taken at baseline for 307 subjects. Individual ADCOMS composite score trajectories were calculated for the same ADNI subjects, using ADAS‐Cog, MMSE, and CDR‐SB assessment raw data over time.

Progression rate for each subject was determined using linear regression. As a further step, each trajectory was partitioned into one of four progression groups using longitudinal clustering methods. These groups were labelled a) Fast, b) Intermediate, c) Slow, and d) None, the last indicating no overall progression.

Spearman rank correlation significance with individual trajectory slope was used to down‐select the 83 biomarkers. The resulting candidate list of 21 biomarkers were assessed for Pearson correlation with progression group membership and adjusted for false discovery rate. This resulted in a priority list of 7 biomarkers used in GLM Logistic regression models to predict progression group membership, and to calculate ROC and AUC summary values to distinguish Fast and Intermediate from Slow and no progression, and other group configurations.

**Result:**

Decreased levels of ApoE, CD40, Chromogranin A (CgA), and VEGF, and increased levels of FABP, FGF4, and PPP were significantly correlated with progression rate and group (adjusted p‐value < 0.05). The Area Under the Curve (AUC) values ranged from 0.718 to 0.774 for between 3 and 7 biomarkers, with considerable variability in repeated 5‐fold cross‐validation. These results indicate a significant but moderate ability of CSF immune biomarkers to differentiate between progression trajectories.

**Conclusion:**

Within ADNI’s clinical trial cohort, CSF biomarker levels of inflammation and immune metabolism demonstrated useful predictive power for progression trajectory group membership, prompting further work to refine the modelling to better understand the vascular and metabolic interaction complexities.